# Impact of COVID‐19 disease and vaccination on dermatological immune‐mediated inflammatory diseases atopic dermatitis, psoriasis, and vitiligo: a Target2B! substudy

**DOI:** 10.1111/1346-8138.17664

**Published:** 2025-02-14

**Authors:** Nicoline F. van Buchem‐Post, Wouter Ouwerkerk, Eileen W. Stalman, Koos P. J. van Dam, Luuk Wieske, Marcel W. Bekkenk, Albert Wolkerstorfer, Phyllis Spuls, Annelie H. Musters, Angela L. Bosma, Dirk‐Jan Hijnen, Filip Eftimov, Rosalie M. Luiten, T2B! immunity against SARS‐CoV‐2 study group, Zoé L. E. van Kempen, Eileen W. Stalman, Maurice Steenhuis, Laura Y. L. Kummer, Koos P. J. van Dam, Anja Ten Brinke, S. Marieke van Ham, Taco Kuijpers, Theo Rispens, Filip Eftimov, Luuk Wieske, Joep Killestein, A. J. Vd Kooi, J. Raaphorst, A. H. Koos Zwinderman, M. Löwenberg, A. G. Volkers, G. R. A. M. D'Haens, R. B. Takkenberg, S. W. Tas, M. L. Hilhorst, Y. Vegting, F. J. Bemelman, N. J. M. Verstegen, L. Fernandez, S. Keijzer, J. B. D. Keijser, O. Cristianawati, A. E. Voskuyl, B. Broens, A. P. Sanchez, S. Nejentsev, E. S. Mirfazeli, G. J. Wolbink, L. Boekel, B. A. Rutgers, K. de Leeuw, B. Horváth, J. J. G. M. Verschuuren, A. M. Ruiter, L. van Ouwerkerk, D. van der Woude, Rcf Allaart, Yko Teng, M. H. Busch, E. Brusse, P. A. van Doorn, Mae Baars, Crg Schreurs, W. L. van der Pol, H. S. Goedee, C. A. C. M. van Els, J. de Wit

**Affiliations:** ^1^ Department of Dermatology Netherlands Institute for Pigment Disorders, Amsterdam University Medical Centers, University of Amsterdam, Amsterdam Institute for Immunology & Infectious Diseases Amsterdam Netherlands; ^2^ National Heart Centre Singapore Singapore Singapore; ^3^ Department of Neurology, Amsterdam Neuroscience Amsterdam UMC Amsterdam Netherlands; ^4^ Department of Dermatology Netherlands Institute for Pigment Disorders, Amsterdam University Medical Centers, VU University, Amsterdam Institute for Infection and Immunity Rotterdam Netherlands; ^5^ Department of Dermatology Erasmus University Rotterdam Rotterdam Netherlands

**Keywords:** atopic dermatitis, COVID‐19, psoriasis, vitiligo

## Abstract

During the COVID‐19 pandemic, the daily life of many patients with dermatological immune‐mediated inflammatory diseases (DIMIDs), such as atopic dermatitis (AD), psoriasis, and vitiligo, was impacted by social restrictions caused by (fear of) morbidity, mortality associated with COVID‐19, and vaccine hesitancy. This prospective observational, multicenter, multidisciplinary cohort study explored the impact of COVID‐19 disease and vaccination on DIMIDs, specifically AD, psoriasis, and vitiligo. Data from patients with DIMIDs were collected as part of the Target2B! study (between February 2021 and October 2022). We analyzed the differences in baseline characteristics, risk of developing COVID‐19, proportion of DIMIDs in patients reaching seroconversion upon vaccination per DIMID, and self‐reported increase in DIMID activity by multivariable logistic regression and sensitivity analyses. A total of 424 patients with DIMID were included. COVID‐19 disease commonly occurred in patients with vitiligo (51.1%), AD (42.0%), and psoriasis (34.3%) (*p* = 0.038). COVID‐19 was not associated with the use of immunosuppressive therapy. Three patients (two with AD and one with vitiligo) were hospitalized due to COVID‐19. Nearly all patients with DIMIDs exhibited effective seroconversion after regular vaccination regimens (vitiligo 100%, psoriasis 97.9%, AD 96.5%). Increased DIMID activity after COVID‐19 (6.6%) or severe acute respiratory syndrome–related coronavirus (SARS‐CoV‐2) vaccination (12.26%) was reported in a minority of patients, with baseline progressive disease (disease activity 3 months preceding baseline survey) being the only associated risk factor (COVID‐19: odds ratio [OR], 4.27 [*p* = 0.02]; vaccination OR, 3.45 [*p* = 0.002]). In conclusion, no alarming signs were shown in this study regarding (severe) COVID‐19 in patients with AD, psoriasis, or vitiligo. Vaccination against COVID‐19 is advised in patients with DIMIDs. Moreover, patients with DIMIDs can safely continue their immunosuppressant therapy, since this does not increase the risk of COVID‐19, while vaccination‐induced humoral responses are adequate. In only a minority of patients, increased DIMID activity after COVID‐19 or SARS‐CoV‐2 vaccination occurred.

## 1 INTRODUCTION

During the COVID‐19 pandemic, the daily life of many patients with dermatological immune‐mediated inflammatory diseases (DIMIDs), such as atopic dermatitis (AD), psoriasis, and vitiligo, was impacted by social restrictions due to (fear of) morbidity, mortality associated with COVID‐19, and vaccine hesitancy. In the general population, before vaccination, 40.5% of the infected people remained asymptomatic, while approximately 33% of hospitalized patients developed acute respiratory distress syndrome.^1^, ^2^ Older age, non‐White ethnicity, male sex, and immunodeficiency diseases are associated with an increased risk of COVID‐19 and severe clinical course and outcome.^3^, ^4^, ^5^, ^6^ Vaccination against severe acute respiratory syndrome–related coronavirus (SARS‐CoV‐2), a healthy diet, sufficient nutrition, and atopic conditions are considered as protective factors against COVID‐19.^7^

The T2B! (Target2B!) study^8^ is a national prospective observational multicenter, multidisciplinary cohort study in patients with immune‐mediated inflammatory diseases (IMIDs). The findings revealed that most patients with IMIDs receiving immunosuppressants (ISPs) show equivalent seroconversion to controls.^8^ Moreover, a minority of patients experienced increased disease activity, as measured by patient‐reported outcomes.^9^ However, in these studies, a large group of the patients with DIMIDs were categorized as controls, since many patients with DIMIDs did not use ISP therapy.

AD, psoriasis, and vitiligo are chronic inflammatory skin diseases, while vitiligo is also autoimmune mediated. In these skin diseases, both genetic and environmental factors play a role in the pathogenesis.^10^, ^11^, ^12^, ^13^ During the pandemic, patients with AD were associated with lower life satisfaction and health rating.^14^

Multiple studies have been performed to investigate the risk of COVID‐19 development in patients with AD^15^, ^16^, ^17^, ^18^, ^19^ and those with psoriasis.^16^, ^20^, ^21^, ^22^ They have shown that patients with AD were more likely to develop COVID‐19^15^, ^16^ and have similar COVID‐19 infection rates^17^ and hospitalization risks compared with controls.^18^ Moreover, the risk of COVID‐19 complications appeared to be low in patients with AD who are treated with systemic immunomodulatory agents.^19^ In psoriasis, an increased risk of COVID‐19 has been shown.^16^, ^20^, ^21^ Moreover, the genetic risk of psoriasis was associated with increased predisposition to COVID‐19.^20^ An electronic survey study^22^ concluded that no difference was found in the severity of COVID‐19 among patients with AD, chronic urticaria, psoriasis, and vitiligo. No studies assessing the risk of COVID‐19 in vitiligo were performed.

The incidence of DIMID progression and its risk factors have not yet been investigated in regards to COVID‐19 and SARS‐CoV‐2 vaccination. Multiple case reports have been published describing patients who acquired psoriasis or vitiligo after developing COVID‐19 and vaccination.^23^, ^24^, ^25^, ^26^ Moreover, cases of disease progression of DIMIDs after COVID‐19 and vaccination have also been reported.^27^, ^28^, ^29^, ^30^, ^31^

Our study examined three objectives: (1) the impact of DIMIDs on COVID‐19 risk and COVID‐19 disease course; (2) the effect of DIMIDs and immunosuppression on SARS‐CoV‐2 vaccination response; and (3) the impact of COVID‐19 or SARS‐CoV‐2 vaccination on DIMIDs activity.

## 2 METHODS

### 2.1 Study design and participants

We performed a substudy of the ongoing T2B! study by analyzing T2B! study participants 18 years and older with DIMIDs (AD, psoriasis, or vitiligo) included between February 2, 2021, and August 1, 2021.^8^ T2B! inclusion was based on IMIDs, use of ISPs, and COVID‐19 development.^8^ The participants were required to understand the questionnaires in Dutch. Participants with known pregnancy during study entry and those undergoing concomitant treatment with ISPs (i.e. chemotherapy) for cancer or organ transplantation (including stem cell transplantation) were excluded. The T2B! study was approved by the medical ethical committee (NL74974.018.20 and EudraCT 2021–001102–30) and registered in the Dutch Trial Register (trial ID NL8900). All participants provided signed informed consent. A detailed description of the T2B! study protocol was previously published.^8^

### 2.2 Procedures

A standardized electronic case report form and online patient‐reported questionnaires were used to collect clinical data in Castor EDC (Netherlands), from March 2020 to October 2022. The questionnaires recorded demographics, dates of any positive polymerase chain reaction results for SARS‐CoV‐2 (to confirm COVID‐19), dates of vaccinations, and increases in DIMID activity. The investigators manually registered immune‐mediated inflammatory disorder diagnosis from the electronic patient files in the electronic case report form.

Changes in disease activity were registered on a five‐point Likert scale. Increased DIMIDs activity was reported when patients noted “worse” and “much worse” response.

Different waves of SARS‐CoV‐2 were registered per time frame in which one of the variations were dominant. The Alpha wave occurred from the beginning of the study up to June 10, 2021; the Delta wave from June 11, 2021, to December 15, 2021; and the Omicron wave from December 16, 2021, until the end of this study.

### 2.3 Serum analyses

The presence of SARS‐CoV‐2 antibodies were assessed by measuring anti–spike protein receptor–binding domain (anti‐RBD) IgG titers in serum samples collected at baseline (before the first vaccination) and 28 days after each vaccination, of two vaccinations in total (if applicable). Serum was collected in special tubes (Minicollect 450 548, Greiner Bio‐One) using an at‐home fingerprick set and analyzed by the Sanquin National Screening Laboratory. Seroconversion after vaccination was defined as an antibody concentration of >4 AU/mL (99% specificity in prepandemic sera).^32^, ^33^

### 2.4 Vaccination

Vaccination of the participants occurred either through the national vaccination campaign or as part of the T2B! study. Completed vaccination against SARS‐CoV‐2 was defined as two vaccinations of the same type for the ChAdOx1 nCoV‐19 (Oxford–AstraZeneca), BNT162b2 (Pfizer–BioNtech), and CX‐024414 (Moderna) vaccines, regardless of the interval, and one vaccination for Ad.26.COV2.S (Janssen). A previous SARS‐CoV‐2 infection was defined as a self‐reported positive PCR for SARS‐CoV‐2 with or without evidence of anti‐RBD antibodies at baseline and follow‐up. In this cohort, primary immunizations ranged from March 1, 2021, to December 10, 2021.

### 2.5 Outcomes and statistical analysis

For baseline characteristics, we reported descriptive values as means±standard deviations (SDs) for continuous variables and as numbers and percentages for ordinal variables (Table 1). We tested the difference in these variables between the AD, psoriasis, and vitiligo groups using analysis of variance or χ^2^ tests.

**TABLE 1:** Baseline characteristics.

Characteristic	All participants	AD	Psoriasis	Vitiligo	*p*‐Value
Number (%)	424 (100%)	176 (30.14%)	70 (11.99%)	178 (30.48%)	NA
Mean age (±SD), years	44.2 (±12.3)	43.5 (±13.7)	49.1 (±13.5)	43.2 (±12.1)	0.19
Sex, male	207 (48.82%)	89 (50.57%)	42 (60.0%)	76 (42.70%)	0.041*
Mean BMI (±SD), kg/m^2^	25.74 (±4.5)	25.80 (±4.7)	28.36 (±5.24)	24.68 (±3.49)	<0.0001*
Progressive disease at baseline	168 (39.62%)	91 (51.70%)	22 (31.43%)	55 (30.90%)	0.0001*
Influenza vaccination	186 (43.87%)	89 (50.57%)	53 (75.71%)	44 (24.72%)	<0.0001*
Use of immunosuppressants	262 (61.8%)	175 (99.4%)	70 (100%)	17 (9.6)	1
Comorbidities					
Diabetes	12 (2.83%)	3 (1.70%)	4 (5.71%)	5 (2.81%)	0.23
Hypertension	25 (5.9%)	10 (5.68%)	10 (14.29%)	5 (2.81%)	0.0025*
Cardiovascular disease	26 (6.13%)	14 (7.95%)	5 (7.14%)	7 (3.93%)	0.027*
Pulmonary disease	45 (10.61%)	35 (19.89%)	4 (5.71%)	6 (3.37%)	<0.0001*
Chronic kidney disease	0 (0%)	0 (0%)	0 (0%)	0 (0%)	1
COVID‐19	189 (44.6%)	74 (42.0%)	24 (34.3%)	91 (51.1%)	0.038*

* *p*=0.05 considered significant. Values are expressed as number (percentage) unless otherwise indicated. AD, atopic dermatitis; BMI, body mass index; SD, standard deviation.

For our primary aim, we reported the risk of developing COVID‐19 between DIMIDs using descriptive statistics as numbers and percentages. The proportion of patients with DIMIDs who developed COVID‐19 was calculated with corresponding 95% confidence intervals (CIs). Moreover, multivariable logistic regression analysis was performed to analyze the confounding factors (Table 2).

**TABLE 2:** Detailed description of used immunosuppressants.

Characteristic	AD	Psoriasis	Vitiligo
Adalimumab	0	12	0
Azathioprine	1	0	0
Baricitinib	7	0	0
Certolizumab	0	1	0
Ciclosporin	37	3	1
Dexamethasone	0	0	2
Dupilumab	148	1	1
Hydroxychloroquine	2	0	1
Leflunomide	0	1	0
Mesalazine	0	0	2
Methotrexate	29	28	1
Mycophenolate mofetil	7	0	1
Other	2	2	2
Predniso(lo)ne	7	1	1
Tacrolimus	0	0	4
Teriflunomide	0	0	1
Ustekinumab	0	40	0

Abbreviation: AD, atopic dermatitis.

For the second aim, we calculated the proportion of patients with DIMIDs who reached seroconversion for all groups. Moreover, we performed sensitivity analysis to investigate the potential influencing factors (age, sex, body mass index [BMI], influenza vaccination, self‐reported increased disease activity in the 3 months preceding the baseline survey, use of ISPs, and comorbidities [diabetes, hypertension, cardiovascular disease, pulmonary disease, chronic kidney disease]) on the titers after vaccination.

For the third aim, all self‐reported increases in disease activity were recovered per wave. These increases were linked to COVID‐19 and/or SARS‐CoV‐2 vaccination only if they occurred within a timeframe of <3 months after these events. The incidence of patients with self‐reported increased disease activity after COVID‐19 or SARS‐CoV‐2 vaccination was calculated, along with the corresponding 95% CIs. Differences in the incidence of self‐reported increased disease activity between DIMID diagnoses, COVID‐19, and vaccines were assessed using χ^2^ test. If a patient presented to a physician because of an increase in disease activity, this was considered as treatment intensification.

A multivariable logistic regression model was performed to investigate the impact of potential factors on developing increased DIMID activity after COVID‐19 and vaccination. Factors that were considered included: age; sex; BMI^3^, ^4^, ^5^, ^6^; yearly vaccination for influenza; diabetes; hypertension; cardiovascular, pulmonary, or kidney disease; self‐reported increased disease activity in the 3 months preceding the baseline survey; and use of ISPs during primary immunization. Determinants are reported as ORs with associated 95% CIs. Gannt charts were used to display increased disease activity, COVID‐19 development, and SARS‐CoV‐2 vaccinations against time.

Data analysis was performed with R version 4.1.0 (R Foundation for Statistical Computing).

## 3 RESULTS

### 3.1 Baseline characteristics

In total, 424 participants with DIMIDs were included in this study, comprising 176 patients with AD (30.14%), 70 patients with psoriasis (11.99%), and 178 patients with vitiligo (30.48%). Of the participants, 207 (48.8%) were male. The participants had a mean age of 44.2± 12.3 years and a mean BMI of 25.74± 4.5. Moreover, 168 patients with DIMIDs reported progressive disease at baseline (self‐reported increased disease activity in the 3 months preceding the baseline survey) (39.3%). Interestingly, almost all (99.4%) and all (100%) patients with AD and psoriasis used ISPs, compared with 9.6% of patients with vitiligo. The patients with DIMIDs showed multiple comorbidities (Table 1). A more detailed overview of the baseline characteristics is shown in Table 1 and ISP details are shown in Table 2.

### 3.2 Impact of DIMIDs on COVID‐19 risk and the COVID‐19 disease course

In total, patients with vitiligo (*n* = 91, 51.1%) developed COVID‐19 significantly more frequently than patients with AD (*n* = 74, 42.0%) and those with psoriasis (*n* = 24, 34.3%) (*p* = 0.038) (Table 3). COVID‐19 incidence was lower in older patients (OR, 0.97; *p*<0.001). Sex or BMI did not significantly influence the risk of developing COVID‐19. Progressive DIMID at baseline was associated with an increased risk of developing COVID‐19 (OR, 1.63 [95% CI, 1.06–2.52], *p* = 0.028). Receiving ISP therapy was not associated with developing COVID‐19 (OR, 1.43 [95% CI, 0.42–5.38], *p* = 0.57). Moreover, participants with lung diseases as a comorbidity developed COVID‐19 less frequently (OR, 0.47; *p* = 0.048). All other comorbidities, including diabetes, hypertension, cardiovascular disease, and chronic kidney disease, did not influence the risk of developing COVID‐19 (Table 3). In total, three patients (two with AD and one with vitiligo) reported to have been hospitalized for COVID‐19 (0.7%). The patient with vitiligo was also admitted to the intensive care unit for 15 days (0.2%) for superinfections, and was diagnosed with a primary immunodeficiency caused by a toll‐like receptor 8 mutation.

**TABLE 3:** Sensitivity analysis of COVID‐19 risk and confounding factors compared with AD.

COVID	OR	LL	UL	*p*‐Value
AD psoriasis*	0.8		1.56	0.51
AD vitiligo*	1.68	1.04	2.73	0.036*
Age	0.97	0.95	0.98	<0.001*
Sex	0.86	0.57	1.31	0.49
BMI	1.03	0.99	1.09	0.17
Influenza vaccination	1.3	0.82	2.09	0.27
Baseline increase DIMID activity	1.63	1.06	2.52	0.028*
Comorbidities				
Diabetes	1.56	0.39	6.01	0.51
Hypertension	0.33	0.09	0.97	0.062
Cardiovascular disease	1.64	0.68	3.97	0.27
Pulmonary disease	0.47	0.22	0.98	0.048*
Chronic kidney disease	7.7	NA	NA	0.12
Use of immunosuppressants	1.43	0.42	5.38	0.57

* *p*=0.05 considered significant. AD, atopic dermatitis; BMI, body mass index; DIMID, dermatological immune‐mediated inflammatory disease; LL, lower limit; NA, not available; OR, odds ratio; UL, upper limit.

The COVID‐19 pandemic consisted of three waves with different COVID‐19–dominant variances. During the Alpha‐dominant wave (up to June 6, 2021), none of the participants with DIMIDs developed COVID‐19. During the Delta‐dominant wave (June 6, 2021, up to December 15, 2021), 14 patients with AD (8.0%), three patients with psoriasis (4.3%), and 19 patients with vitiligo (10.7%) had COVID‐19. No significant differences between the DIMID groups were found (*p* = 0.25). COVID‐19 was reported mostly during the Omicron‐dominant wave (from December 15, 2021). In total, 49 patients with AD (27.8%), 15 patients with psoriasis (21.4%), and 69 patients with vitiligo (38.8%) were diagnosed with COVID‐19. Patients with vitiligo developed COVID‐19 with the Omicron variant significantly more frequently (*p* = 0.012).

### 3.3 Impact of DIMIDs on SARS‐CoV‐2 vaccination response

**FIGURE 1:**
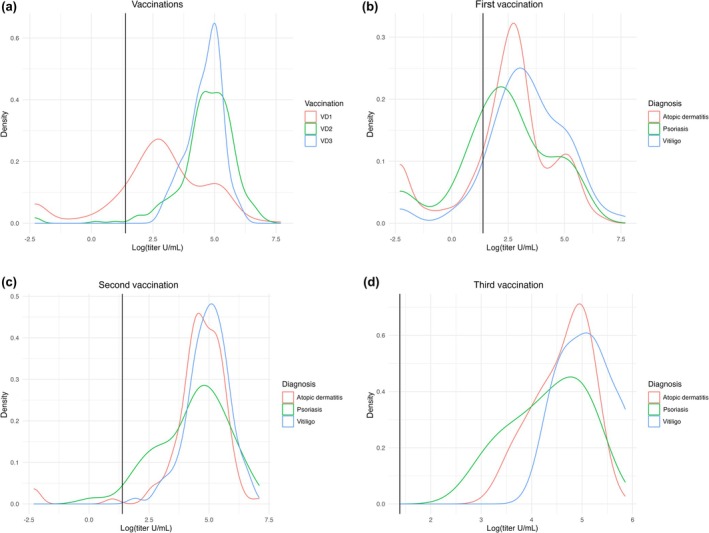
(a) Antibody concentration after severe acute respiratory syndrome–related coronavirus (SARS‐CoV‐2) vaccinations. (b–d) Antibody concentration after the first, second, and third vaccinations. Density = number of participants. *4 AU/mL mark is illustrated with black bar (clinically significant seroconversion at 4 AU/mL).

To calculate the proportion of seroconversion, we distinguished the various types of vaccinations per DIMID. There was a significant difference in the distribution of first vaccine type (Table 4). Whereas patients with AD received mostly Moderna or Pfizer–BioNtech, patients with psoriasis most often received the AstraZeneca vaccine, and patients with vitiligo more often received the Janssen vaccine. It is of importance to state that almost all of the participants (69.0% AD, 78.6% psoriasis, and 91.1% vitiligo) already showed a positive seroconversion titer above the clinically significant level of 4 AU/mL after the first vaccination (Figure 1). After the second vaccination, 96.5% of patients with AD, 97.9% of patients with psoriasis, and 100% of patients with vitiligo reached seroconversion.

**TABLE 4:** Different types of vaccinations per DIMID group and number of vaccinated participants.

	All participants, *N* (%)	AD, *n* (%)	Psoriasis, *n* (%)	Vitiligo, *n* (%)	*p*‐Value
Vaccination					0.01*
AstraZeneca	8 (3%)	2 (2%)	4 (8%)	2 (2%)	
Janssen	16 (5%)	3 (2%)	2 (4%)	11 (10%)	
Moderna	8 (3%)	4 (3%)	3 (6%)	1 (1%)	
Pfizer/BioNTech	109 (37%)	48 (35%)	21 (40%)	40 (37%)	
Moderna or Pfizer	113 (38%)	62 (45%)	18 (34%)	33 (31%)	
First vaccination	327 (55.99%)	117 (66.48%)	49 (70.0%)	86 (48.31%)	0.02*
Second vaccination	277 (47.43%)	105 (59.66%)	45 (64.29%)	65 (36.52%)	0.005*
Third vaccination	32 (5.48%)	14 (7.95%)	10 (14.29%)	8 (4.4%)	0.04*

* *p*=0.05 considered significant. AD, atopic dermatitis; DIMID, dermatological immune‐mediated inflammatory disease.

Overall, older age was associated with significantly lower seroconversion rates after the first or second vaccination (*p* < 0.001), whereas older age was not affected by progressive disease at baseline (*p* = 0.64), baseline immunization (*p* = 0.35), or sex (*p* = 0.80).

### 3.4 Impact of COVID‐19 or SARS‐CoV‐2 vaccination on DIMID activity

**FIGURE 2:**
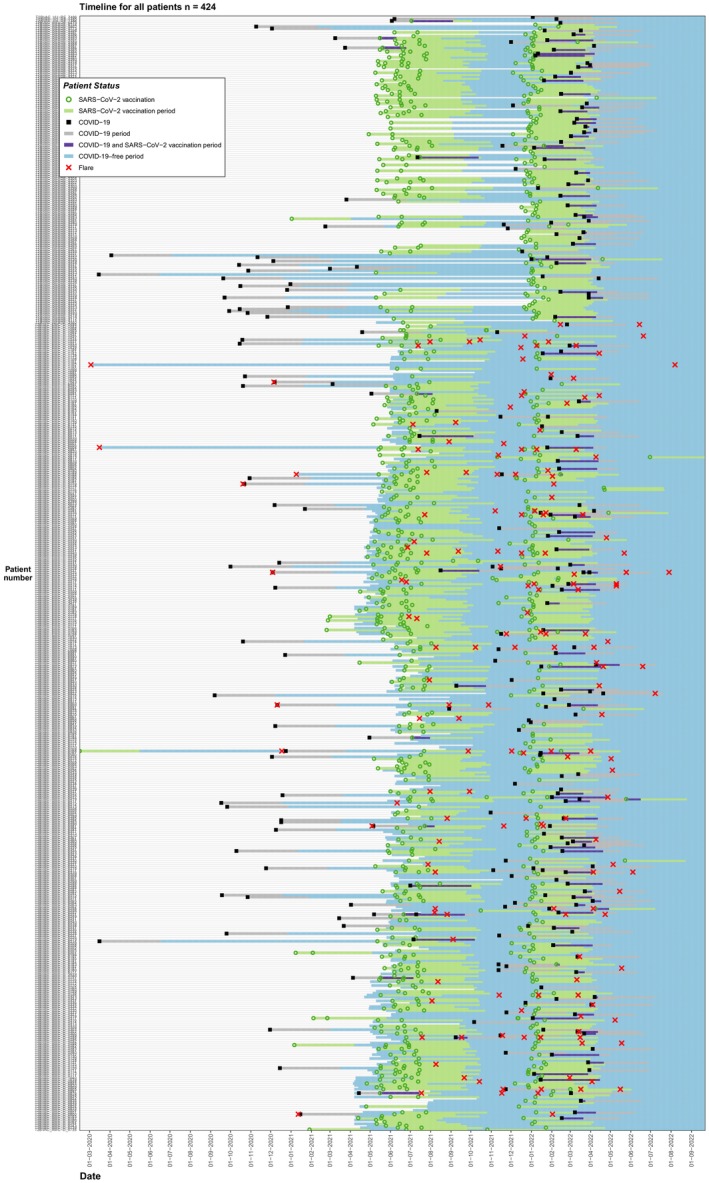
Gantt chart with the timeframe of the study and the reported increased disease activity after COVID‐19, severe acute respiratory syndrome–related coronavirus (SARS‐CoV‐2) vaccination, or outside of these periods in all patients with dermatological immune‐mediated inflammatory diseases (DIMIDs). All the participants (y axis) with registered SARS‐CoV‐2 vaccination dates (green circle), COVID‐19 development dates (black box), and dates of registered DIMID activity (red X). After SARS‐CoV‐2 vaccination and COVID‐19 development, a period of 90 days was taken as the COVID‐19 period (gray line), SARS‐CoV‐2 vaccination period (green line), and COVID‐19 + SARS‐CoV‐2 period (purple line). If an increase in DIMID activity was registered in this period, it was linked to COVID‐19 development or SARS‐CoV‐2 vaccination. Moreover, COVID‐19–free periods are shown with the blue lines. The dashed vertical lines represent the turning points from the Alpha‐dominant to Delta‐dominant wave and the Delta‐dominant to Omicron‐dominant wave, respectively.

In total, increased DIMID activity was reported 384 times (range, 2–13), either after COVID‐19 (*n* = 88, 22.9%), vaccination (*n* = 199, 51.8%), or outside of these periods (*n* = 97, 25.2%) (Figure 2).

Increased disease activity after COVID‐19 was reported in 28 patients with DIMID (6.6% [range, 2–7]) (Table 5). Four psoriasis patients, 12 patients with AD (6.8%), and 12 patients with vitiligo (6.7%) reported increased disease activity at least once [Files S1, S2, S3]. Nine patients consulted a medical specialist due to the increased disease activity, six of these patients reported a treatment change after seeing the specialist.

**TABLE 5:** Number of participants who reported an increase in DIMID activity after SARS‐CoV‐2 vaccination and COVID‐19, and the range of times an increase in DIMID activity was reported.

Self‐reported increase DIMID disease activity	All participants	AD	Psoriasis	Vitiligo
Total, *n* (%) [range times]	80 (18.9%) 2, 3, 4, 5, 6, 7, 8, 9, 10, 11, 12, 13]	27 (21.0%) 2, 3, 4, 5, 6, 7, 8, 9, 10, 11, 12, 13]	16 (22.8%) 2, 3, 4, 5, 6, 7, 8, 9, 10, 11, 12, 13]	27 (15.1%) 2, 3, 4, 5, 6, 7, 8, 9, 10, 11, 12, 13]
After COVID‐19, *n* (%) [range times]	28 (6.6%) [2, 3, 4, 5, 6, 7]	12 (6.8%) 2, 3, 4, 5, 6, 7]	4 (5.7%) 2, 3, 4]	12 (6.7%) [2, 3, 4, 5]
After SARS‐CoV‐2 vaccination, *n* (%) [range times]	52 (12.3%) 2, 3, 4, 5, 6, 7, 8, 9, 10, 11, 12, 13]	25 (14.2%) 2, 3, 4, 5, 6, 7, 8, 9, 10, 11, 12, 13]	12 (17.1%) [2, 3, 4, 5, 6, 7, 8, 9, 10]	25 (8.4%) 2, 3, 4, 5, 6, 7]

Abbreviations: AD, atopic dermatitis; DIMID, dermatological immune‐mediated inflammatory disease; SARS‐CoV‐2, severe acute respiratory syndrome–related coronavirus.

Increased disease activity after the SARS‐CoV‐2 vaccinations was reported in 52 patients (12.26% [range, 2–13]). Twelve patients with psoriasis (17.1%), 25 patients with AD (14.2%), and 15 patients with vitiligo (8.4%) reported increased disease activity at least once after the SARS‐CoV‐2 vaccination (Files S1, S2, S3). Nineteen patients were seen by a medical specialist because of increased disease activity after the SARS‐CoV‐2 vaccination, and in seven patients it was known that their treatment increased or changed.

Progressive disease at baseline was the only risk factor associated with reporting increased DIMID activity after COVID‐19 (OR, 4.27; *p* = 0.02) or SARS‐CoV‐2 (OR, 3.45; *p* = 0.002) vaccination (Table 3). In patients with DIMIDs with a stable disease course, no increase in DIMID activity was seen after COVID‐19 and SARS‐CoV‐2 vaccination.

## 4 DISCUSSION

This prospective observational multicenter and multidisciplinary cohort showed that patients with vitiligo have a higher risk of developing COVID‐19, as compared with patients with AD and those with psoriasis. Only three patients (two with AD and one with vitiligo) were hospitalized for COVID‐19; the other participants did not develop severe COVID‐19. Moreover, patients with DIMIDs can safely continue ISP therapy, since this does not affect the risk of developing COVID‐19. Nearly all patients with DIMIDs showed positive seroconversion levels on regular vaccination regimens. Self‐reported increased disease activity after COVID‐19 and SARS‐CoV‐2 vaccination was seen in only a minority of the patients. An increase in DIMID activity at baseline (disease activity 3 months preceding the baseline survey) was the only associated risk factor.

According to the data of the Dutch National Institute for Public Health and the Environment,^34^ patients with vitiligo were at higher risk of COVID‐19 both in the Delta‐ and Omicron‐dominant waves (10.67% and 38.76%, respectively), compared with the general Dutch population (Delta 4.98%, Omicron 29.84%). Even though this study was conducted in an academic specialized hospital where patients with severe skin diseases and ISP therapy are frequently seen, the COVID‐19 risk in patients with AD (Delta 7.95%, Omicron 27.84%) and psoriasis (Delta 4.29%, Omicron 21.43%) was comparable to the Dutch population for both the Delta‐ and Omicron‐dominant waves (4.98% and 29.84%, respectively). It could be speculated that the increased risk in patients with vitiligo is due to their use of fewer ISPs and therefore taking fewer precautionary measures against SARS‐CoV‐2 virus compared with patients with AD and psoriasis.

One questionnaire study investigated the severity of COVID‐19 in patients with AD, psoriasis, or vitiligo.^22^ They found no difference in the severity of COVID‐19 between the skin diseases. Moreover, Rakita et al. also found that AD is not an independent risk factor for COVID‐19 severity or complications.^18^ This study found that three patients (two with AD and one with vitiligo) were hospitalized for COVID‐19, of whom the patient with vitiligo was also admitted to the intensive care unit. In these patients, COVID‐19 was mostly mild.

Known risk factors for developing COVID‐19, such as BMI and male sex, did not influence the risk of developing COVID‐19 in our study population. However, interestingly, since older age was a protective factor for developing COVID‐19, it could be speculated that older individuals were more cautious with social interactions and practiced greater physical distancing. Regarding the use of ISP medication, Wu et al.^35^ concluded that the use of oral methotrexate and tumor necrosis factor inhibitors did not increase the risk of developing COVID‐19 in patients with psoriasis compared with the general population. Whereas another study showed that an increased odds of COVID‐associated hospitalization was associated with the use of oral methotrexate for psoriasis.^36^ Moreover, the risk of COVID‐19 complications appear to be low in patients with AD who are treated with systemic immunomodulatory agents.^19^ Our study also showed no association between ISP therapy and developing COVID‐19; however, ISPs were not differentiated.

In this study, almost all patients with DIMIDs showed positive seroconversion levels above the clinically significant level of 4 AU/mL after regular vaccination regimens. Two other studies that investigated seroconversion were performed in patients with psoriasis. A prospective study with 77 patients with psoriasis showed high anti–SARS‐CoV‐2‐S IgG seroconversion rates.^37^ Moreover, another prospective study with 101 patients with psoriasis and 55 controls showed no differences in the median serum level of anti–SARS‐CoV‐2S antibody.^38^ Mahil et al. showed that patients with psoriasis who received methotrexate reached lower seroconversion rates with a single dose of BNT162b2 (Pfizer‐BioNTech).^39^ In our study, only a small proportion of the patients with psoriasis did not reach seroconversion after one dose, which diminished after completing the regular vaccination regimens of two doses of BNT162b2.

Increased DIMID activity after developing COVID‐19 and SARS‐CoV‐2 vaccination in patients with DMIDs was seen in only a minority of the patients. Most of these patients reported increased disease activity at least once. Preexisting progressive DIMID activity was found to be an associated risk factor for self‐reported increased DIMID activity after COVID‐19 and/or SARS‐CoV‐2 vaccination. A stable DIMID course showed no increase in DIMID activity after COVID‐19 or after SARS‐CoV‐2 vaccination. Moreover, it is reassuring that only a few patients needed to be referred to a specialist. To date, no other prospective data have been published showing the influence of COVID‐19 and/or SARS‐CoV‐2 vaccination on DIMID activity specifically.

To our knowledge, this is the first study to investigate the incidence of increased disease activity after COVID‐19 development and vaccination in patients with DIMIDs such as AD, psoriasis, and vitiligo.

Our study has some limitations. This study contained an enriched COVID‐19–positive healthy control group, which impeded a risk analysis with the control group. Due to the large heterogeneity in ISPs used and vaccination types, statistical analysis could not be performed. Moreover, disease activity after COVID‐19 and/or SARS‐CoV‐2 vaccination was self‐reported, and, given the nature of the study, possibly overreported, although we used therapeutic changes as a tool to objectify these reports.

### 4.1 Take‐home messages

The current study provides insights into the risk of developing COVID‐19, increased disease activity after COVID‐19 and/or SARS‐CoV‐2 vaccination, and seroconversion rates in patients with the DIMIDs AD, psoriasis, and vitiligo.

No alarming signs were observed in this study regarding the risk of developing (severe) COVID‐19 in patients with AD, psoriasis, and vitiligo. Vaccination against COVID‐19 is advised in patients with DIMIDs, since effective seroconversion levels are reached after regular vaccination regimens. Moreover, patients with DIMIDs can safely continue their immunosuppressant therapy, since this does not increase the risk of COVID‐19 while vaccination‐induced humoral responses were adequate. In only a minority of patients, increased DIMID activity after COVID‐19 or SARS‐CoV‐2 vaccination occurred.

## FUNDING INFORMATION

This study was funded by ZonMw (The Netherlands Organization for Health Research and Development; project number 10430012010009). There was no role in the design, analyses, or reporting of the study by the sponsor.

## CONFLICT OF INTEREST STATEMENT

P. Spuls has received departmental independent research grants for the TREAT NL (Treatment of Atopic Eczema, The Netherlands) registry from Pharma since December 2019; is involved in performing clinical trials with many pharmaceutical industries that manufacture drugs used for the treatment of psoriasis and AD, for which financial compensation is paid to the department/hospital; and is chief investigator of the systemic and phototherapy atopic eczema registry (TREAT NL) for adults and children and one of the main investigators of the SECURE‐AD (Surveillance Epidemiology of Coronavirus (COVID‐19) Under Research Exclusion) registry. F. Eftimov reports (governmental) grants from ZonMw to study immune response after SARS‐CoV‐2 vaccination in autoimmune diseases.

## Supporting information


File S1.

File S2.

File S3.

## Data Availability

The data are available on request from the authors.
