# Supplementary figures and images for: Impact of COVID‐19 disease and vaccination on dermatological immune‐mediated inflammatory diseases atopic dermatitis, psoriasis, and vitiligo: a Target2B! substudy
Source: J Dermatol. 2025 Feb 14;52(4):624–33. doi: 10.1111/1346-8138.17664 (PMC11975183; doi:10.1111/1346-8138.17664)

Treatment Timeline for N=175 Patients

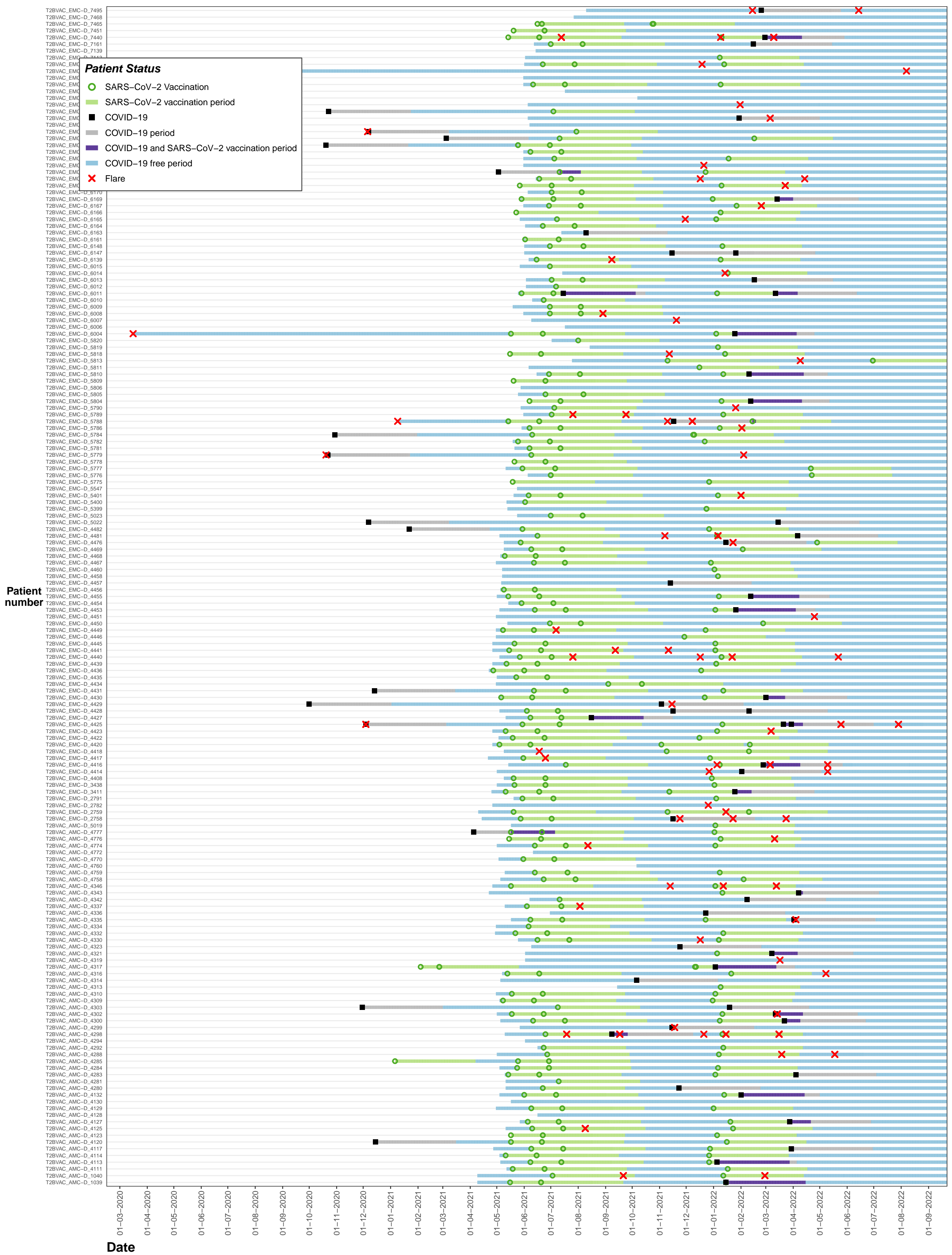

Supplement: Supplementary file 1 — File S1. [file JDE-52-624-s002.pdf]

Treatment Timeline for N=69 Patients

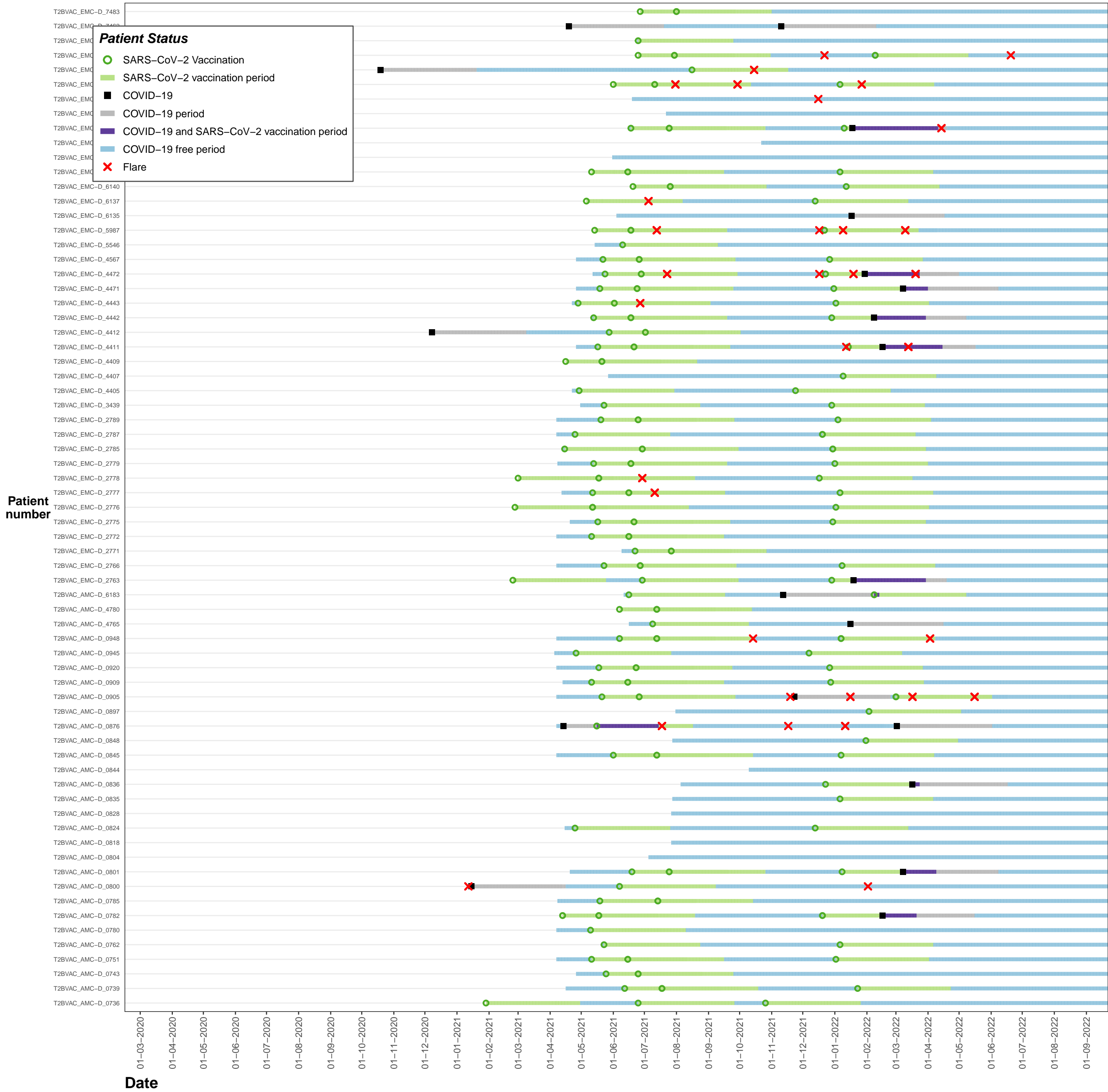

Supplement: Supplementary file 2 — File S2. [file JDE-52-624-s001.pdf]
