# Supplementary material for: Impact of COVID‐19 disease and vaccination on dermatological immune‐mediated inflammatory diseases atopic dermatitis, psoriasis, and vitiligo: a Target2B! substudy
Source: J Dermatol. 2025 Feb 14;52(4):624–33. doi: 10.1111/1346-8138.17664 (PMC11975183; doi:10.1111/1346-8138.17664)
Supplement: Supplementary file 3 — File S3. [file JDE-52-624-s003.pdf]

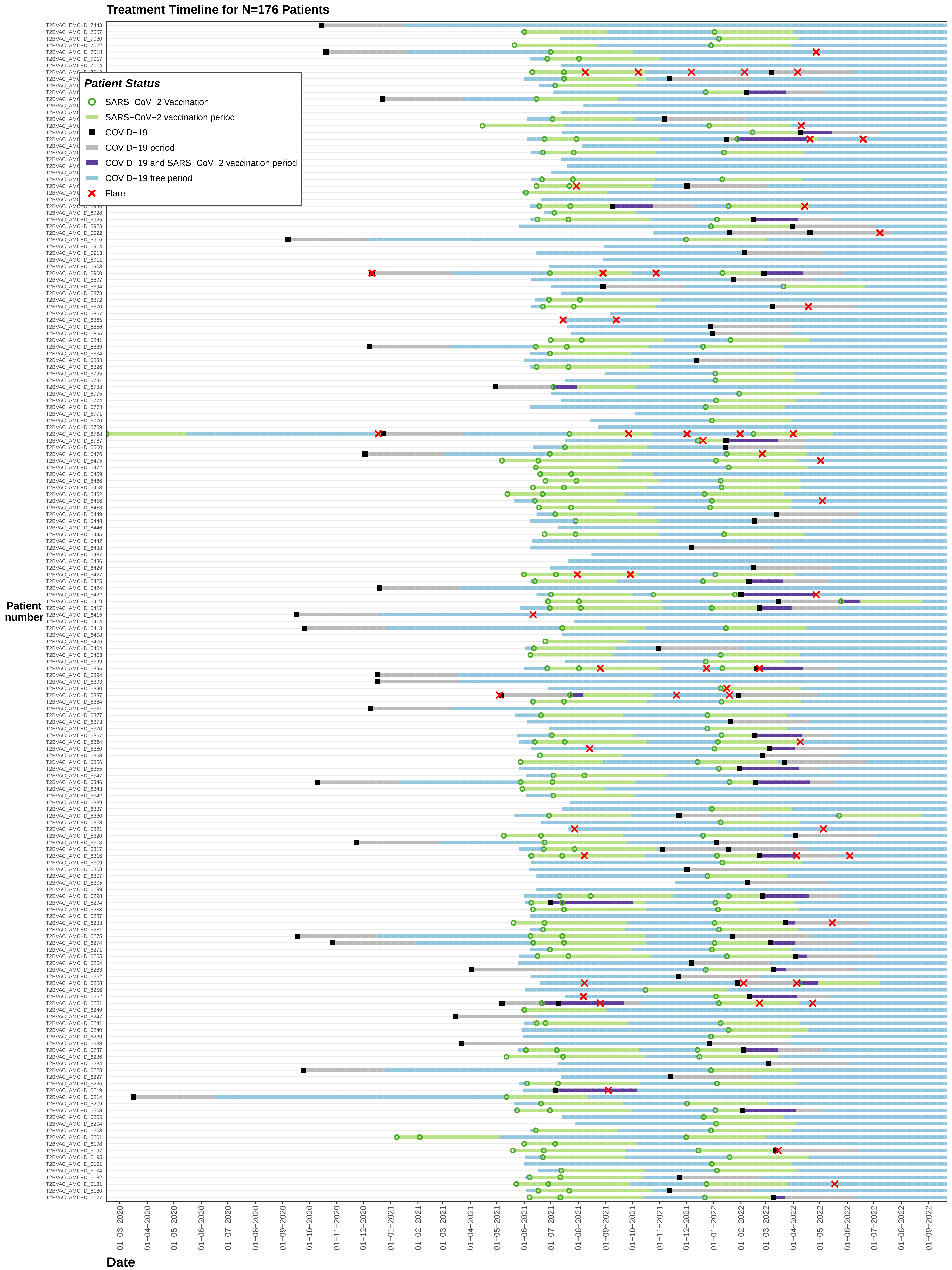
Treatment Timeline for N=176 Patients
Patient Status
SARS−CoV−2 Vaccination
SARS−CoV−2 vaccination period
COVID−19
COVID−19 period
COVID−19 and SARS−CoV−2 vaccination period
COVID−19 free period
Flare
Patient number
Date
01−03−2020
01−04−2020
01−05−2020
01−06−2020
01−07−2020
01−08−2020
01−09−2020
01−10−2020
01−11−2020
01−12−2020
01−01−2021
01−02−2021
01−03−2021
01−04−2021
01−05−2021
01−06−2021
01−07−2021
01−08−2021
01−09−2021
01−10−2021
01−11−2021
01−12−2021
01−01−2022
01−02−2022
01−03−2022
01−04−2022
01−05−2022
01−06−2022
01−07−2022
01−08−2022
01−09−2022
